# Alterations of Apolipoprotein A1, E, and J Genes in the Frontal Cortex in an Ischemic Model of Alzheimer’s Disease with 2-Year Survival

**DOI:** 10.3390/ijms27010326

**Published:** 2025-12-28

**Authors:** Ryszard Pluta, Marzena Ułamek-Kozioł, Janusz Kocki, Anna Bogucka-Kocka, Jacek Bogucki, Stanisław J. Czuczwar

**Affiliations:** 1 Department of Pathophysiology, Medical University of Lublin, 20-090 Lublin, Poland; czuczwarsj@yahoo.com; 2 Department of Neurology, Institute of Psychiatry and Neurology, 02-957 Warsaw, Poland; marzena_ulamek@wp.pl; 3 Department of Clinical Genetics, Medical University of Lublin, 20-080 Lublin, Poland; janusz.kocki@tlen.pl; 4 Department of Biology and Genetics, Medical University of Lublin, 20-093 Lublin, Poland; anna.kocka@tlen.pl; 5 Faculty of Medicine, John Paul II Catholic University of Lublin, 20-708 Lublin, Poland; jacekbogucki@wp.pl

**Keywords:** brain ischemia, Alzheimer’s disease, apolipoprotein A1, apolipoprotein E, apolipoprotein J, clusterin, frontal cortex, genes

## Abstract

In this article, we present genetic studies of apolipoproteins associated with Alzheimer’s disease in the frontal cortex after ischemia and discuss their involvement in the development of neurodegeneration. Gene expression was assessed using an RT-PCR protocol at 2, 7, and 30 days and at 6, 12, 18, and 24 months after an episode of 10 min total cerebral ischemia. *ApoA1* expression (encoding apolipoprotein A1) in the ischemic frontal cortex was lower than control values after 2 days, 6 and 12 months, while its overexpression was observed after 7 and 30 days and 18 and 24 months. In the case of *ApoE* (encoding apolipoprotein E) expression, it was lower than control values after 2 and 30 days and after 6 months; in the remaining periods after ischemia, the expression was above control values. A similar expression pattern after ischemia was revealed for *ApoJ* (encoding apolipoprotein J). The data indicate that the observed changes in gene expression may reflect the activation and inhibition of various pathological processes involved in the development of post-ischemia neurodegeneration. Thus, overexpression of *ApoA1* may be associated with the induction of neuroprotective mechanisms, whereas increased expression of *ApoE* may have harmful effects. Regarding the overexpression of *ApoJ*, the data indicate a dual behavior: in the early stages after ischemia, it has a protective effect, whereas in the later stages, it participates in the progression of neurodegenerative processes.

## 1. Introduction

Rising life expectancy and an aging population worldwide are leading to a dramatic increase in the number of people suffering from dementia in late life, which is now the second leading cause of death in high-income countries [1]. The causes of the described situation are brain diseases, leading to neurodegeneration, which drastically limit brain activity and reduce the quality and length of life, posing a serious problem for modern society. These include Alzheimer’s disease and focal or complete brain ischemia, which occupy a special place among the most common causes of disability, dementia and mortality worldwide [1,2,3,4,5,6,7]. Recent studies have shown that Alzheimer’s disease and cerebral ischemia share common pathogenetic processes, such as overlapping genomic and proteomic alterations and molecular mechanisms [2,6,8,9,10,11,12,13].

The early stages of Alzheimer’s disease are usually asymptomatic, with noticeable symptoms appearing gradually and becoming more severe as the disease progresses over a period of about 20 years [10,14,15,16]. Often, people do not realize they have Alzheimer’s disease until they begin to experience mild cognitive impairment. As neurodegenerative processes in the brain progress, the number of symptoms in patients increases, indicating the appearance of advanced, severe and more extensive neuropathological changes. Among many symptoms, patients may experience difficulty performing daily activities, memory impairment, cognitive decline, mood swings, and sleep disturbances. As a result of the neurological and psychological symptoms, people affected by this extremely debilitating and fatal form of dementia often require assistance with activities that would otherwise be considered routine.

Finally, Alzheimer’s disease leads to progressive neuronal death and brain atrophy, resulting in a gradual loss of cognitive abilities and memory, as well as personality and behavioral changes, ultimately leading to complete brain failure and death [17]. A number of pathological processes are observed in the course of Alzheimer’s disease, including amyloid neurotoxicity, tau protein pathology, cholinergic failure, excitotoxicity, mitochondrial dysfunction, autophagy failure, oxidative stress, apolipoprotein abnormalities, and circulatory problems [7,17,18]. Amyloid accumulation triggers downstream events, such as microglia and astrocyte activation, cytokine secretion, neuroinflammatory response, impaired axonal transport, blood–brain barrier permeability, cerebral amyloid angiopathy, intracellular aggregation of hyperphosphorylated tau protein, and synaptic dysfunction [17]. This neuropathology predominates in structures important for episodic memory, such as the hippocampus and the entorhinal cortex [19].

Within the decades of Alzheimer’s disease research, animal models have been an important tool for understanding the etiology of the disease and for testing therapeutic approaches in preclinical studies [20]. Rodents are the most commonly used animals to model Alzheimer’s disease. Therefore, transgenic mouse models have been developed by overexpressing genetic mutations associated with familial Alzheimer’s disease [21,22]. Mouse models typically reveal the most important neuropathological features of Alzheimer’s disease, but none of them reflect all the neuropathological, neurochemical, and behavioral changes associated with this disease [14,23]. Moreover, most mouse models of familial Alzheimer’s disease based on genetic mutations represent extreme cases of the disease that are not typically observed in humans. These models take the amyloid cascade as their starting point, which assumes that amyloid accumulation will lead to the development of Alzheimer’s disease. However, recent research contradicts this hypothesis, claiming that amyloid is not a factor in the disease process [6,23,24]. Rejecting the amyloid theory can have serious consequences, but clinging to an incorrect idea can be even more harmful. Of course, the vast majority of scientists have investigated the amyloid hypothesis from various perspectives, but they have failed to determine what ultimately causes the development of Alzheimer’s disease.

As is well known worldwide, all previous attempts to modify the course of Alzheimer’s disease by eliminating amyloid have failed [23]. The very fact that not every successful removal of amyloid plaques slowed the progression of Alzheimer’s disease indicates that the amyloid plaques themselves have an insignificant impact on the progression of the disease [25]. It is known that amyloid is necessary but not sufficient to cause Alzheimer’s disease-type dementia. Thus, the failure of clinical trials has forced the scientific community to critically evaluate the validity of the assumptions underlying the amyloid theory of Alzheimer’s disease [23]. The facts presented above do not indicate that amyloid plaques do not play a role in Alzheimer’s disease, but they are probably not the sole or direct cause of the disease. There is therefore an urgent need to develop innovative hypotheses or new models regarding how the core neuropathology of Alzheimer’s disease and associated pathways interact to cause and accelerate dementia. So, it is time to put aside hypotheses and look for alternative explanations for the causes of the disease.

We believe that the ultimate explanation for the causes of Alzheimer’s disease will have to include amyloid as an important part of the story. It is important to emphasize that correlation does not always imply causation; namely, neuronal death and amyloid accumulation are the most likely outcomes of the third process. The lack of understanding of the etiology of Alzheimer’s disease is linked to the lack of effective treatments. Therefore, appropriate models that mimic the late onset of Alzheimer’s disease step by step are urgently needed to bring basic research closer to clinical applications. Moreover, although genetic and epigenetic changes in Alzheimer’s disease have been well-recognized, the precise mechanisms by which these alterations impact disease progress remain unclear. Abundant evidence supports the idea that amyloid is an essential element of Alzheimer’s disease neuropathology, and recent studies indicate that amyloid production and accumulation are linked to impaired blood circulation in the brain [6,8,11,26,27,28,29,30,31]. Inadequate circulation and/or stopped focal or complete blood flow to the brain have been shown to occur before the accumulation of amyloid plaques and neurofibrillary tangles [26,27,28,31,32,33]. Inadequate circulation causes amyloid deposition, which affects blood flow in the brain, resulting in long-lasting cerebral hypoperfusion and disruption of the integrity of the blood–brain barrier [31,34,35]. Studies have shown identical neuropathological processes in post-ischemic brain neurodegeneration and Alzheimer’s disease, including amyloid plaques, neurofibrillary tangles, white matter lesions, cerebral amyloid angiopathy, neuroinflammation, and oxidative stress [8,15,16,28,32,33,36]. Ischemia has also been shown to affect acetylcholine levels in the brain [37,38]. The simultaneous occurrence of acute and chronic neuronal cell damage in post-ischemic brains indicates a continuous, slow, but progressive neuronal response to ischemia, ultimately manifesting as neuronal loss and global brain atrophy [8,39,40]. The consequences of post-ischemic neurodegeneration include impairment of cognitive functions, such as learning and memory, including the development of Alzheimer’s disease-type dementia [41,42,43,44]. For these reasons, cerebral ischemia in rats was chosen for the study, as this model enables long-term survival for animals (2 years), which in turn allows for a detailed analysis of the progressive effects of neurodegenerative processes over time.

It has been shown that in different stages of Alzheimer’s disease [45] and cerebral ischemia [6,15,16], different parts of the brain are affected differently. Furthermore, gene changes in different brain regions following ischemia vary depending on context, region, and time [6]. Identifying gene changes in specific brain regions appears crucial for understanding the neuropathological mechanisms of both diseases. Recently, the importance of ischemic elements in Alzheimer’s disease has been increasingly recognized [2,6,10,11,13,15,16]. Apolipoproteins are known to influence several pathways involved in neurodegeneration in Alzheimer’s disease; namely, apolipoprotein E interacts with presenilin [46,47,48]. In addition to its primary role in amyloid generation and deposition, apolipoprotein E also influences tau protein pathology, neurodegeneration, and the microglial response in Alzheimer’s disease [48,49]. The role of apolipoproteins (Apos) in the development of dementia is less known, especially in Alzheimer’s disease and after cerebral ischemia [50,51]. Apos are known to play a key role post-ischemia in neuroinflammation, blood–brain barrier repair, and neuronal regeneration, making them key players in progression and recovery after an ischemic episode [27,39,47,51,52,53,54,55,56,57,58,59,60]. Considering the fact that staining of apolipoproteins A1, E and J in the intra- and extracellular spaces was significantly increased in the post-ischemic brain in the model used [27,39,55], we decided to investigate the expression of the corresponding genes (*ApoA1*, *ApoE* and *ApoJ*), also called clusterin (*CLU*), using PCR in the frontal cortex of rats that survived cerebral ischemia for a period of 2 years.

## 2. Results

### 2.1. Mean Expression Levels of ApoA1 in the Frontal Cortex at Various Periods After Global Brain Ischemia in Rats

**Figure 1:**
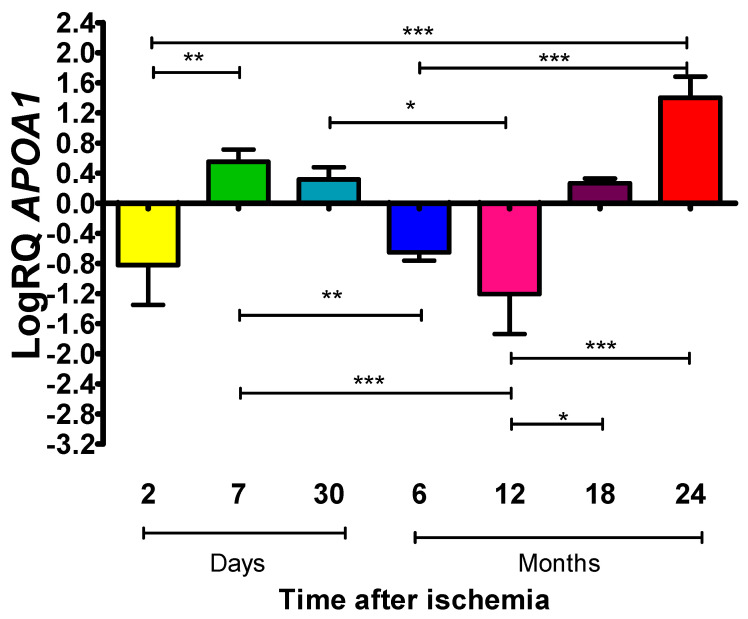
Mean expression levels of *ApoA1* in the frontal cortex. Expression was assessed after 2 (n = 10), 7 (n = 9), and 30 (n = 6) days and after 6 (n = 10), 12 (n = 10), 18 (n = 10), and 24 (n = 10) months after cerebral ischemia. Bars represent standard deviation (SD). The Kruskal–Wallis test was followed by the “z” test for multiple comparisons. * *p* ≤ 0.05, ** *p* ≤ 0.01, *** *p* ≤ 0.001.

The *ApoA1* encodes the apolipoprotein A1. Apolipoprotein A1 is considered a neuroprotective factor in post-ischemic brain neurodegeneration. After 2 days and 0.5 and 1 year following ischemic injury, there was decreased *ApoA1* expression; in other survival times, an opposite trend was noted (Figure 1). All presented data in the figures are presented using a logarithmic formula. Two days after ischemia, the median was −0.788, the minimum was −1.585, the maximum was −0.212, and the mean was −0.822 ± 0.529. Seven days after the ischemic episode, the median was 0.474, the minimum was 0.408, the maximum was 0.946, and the mean was 0.551 ± 0.164. On the 30th day, the median was 0.295-fold, the minimum was 0.139, the maximum was 0.581, and the mean was 0.317 ± 0.162. Half a year after the onset of ischemia, the median was −0.611, the minimum was −0.839, the maximum was −0.551, and the mean was −0.654 ± 0.108. Moreover, 1 and 1.5 years after ischemia, the median was −1.259 and 0.253, respectively. The minimum was −1.959 and 0.189, the maximum was −0.499 and 0.389, and the mean was −1.207 ± 0.531 and 0.266 ± 0.062, respectively. Two years post-ischemia, the median was 1.431, the minimum was 1.021, the maximum was 1.733, and the mean was 1.404 ± 0.278. Figure 1 presents the mean values of *ApoA1* expression and significant differences at various periods following ischemic brain injury.

### 2.2. ApoE Expression Levels in Rat Frontal Cortex Following Global Brain Ischemia

**Figure 2:**
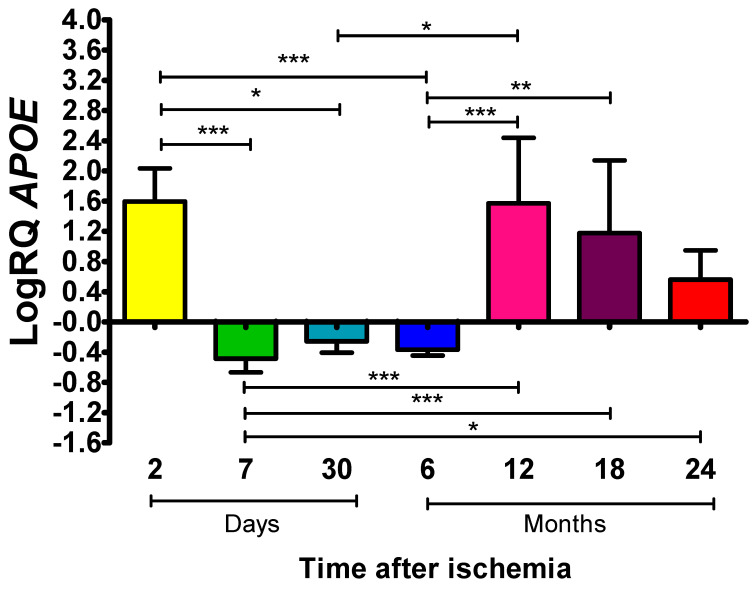
Mean expression levels of *ApoE* in the frontal cortex. Expression was assessed after 2 (n = 10), 7 (n = 9) and 30 (n = 6) days and after 6 (n = 10), 12 (n = 10), 18 (n = 10) and 24 (n = 10) months after cerebral ischemia. Bars represent (SD) standard deviation. The Kruskal–Wallis test was followed by the “z” test for multiple comparisons. * *p* ≤ 0.05, ** *p* ≤ 0.01, *** *p* ≤ 0.001.

The *ApoE* encodes the apolipoprotein E. Apolipoprotein E is thought to have a deleterious effect on the brain following ischemia. On 7 and 30 days and 0.5 years post-ischemia, there was a decline in the expression of *ApoE* below the control level, and during the remaining observation times, it exceeded the control values (Figure 2). *ApoE* expression on day 2 was as follows: the median showed a 1.641-fold increase, the minimum a 1.096-fold increase, the maximum a 2.498-fold increase, and the mean a 1.596 ± 0.438 change. Seven days post-injury, the median showed a −0.530-fold change, the minimum a −0.664-fold change, the maximum a −0.156-fold change, and the mean a −0.486 ± 0.180-fold change. After 30 days and a half a year, the respective values remained below the control: the medians showed a change of −0.291 and −0.341, the minimum changes of −0.406 and −0.521, the maximum changes of −0.061 and −0.296, and the means changes of −0.255 ± 0.151 and −0.368 ± 0.077. A continuous increase in *ApoE* expression was observed after 1, 1.5 and 2 years, and the medians showed a 2.073-fold increase (minimum 0.403 and maximum 2.431), 1.137-fold increase (minimum 0.216 and maximum 2.267), and 0.459-fold increase (minimum 0.156 and maximum 1.266), respectively. At the above-mentioned survival times, the mean expression values were as follows: 1.569 ± 0.870, 1.178 ± 0.961, and 0.562 ± 0.387. Figure 2 presents mean *ApoE* expression values and statistically significant differences between times after the resumption of cerebral circulation.

### 2.3. The Influence of Global Brain Ischemia on the Expression Levels of ApoJ in the Rat Frontal Cortex

**Figure 3:**
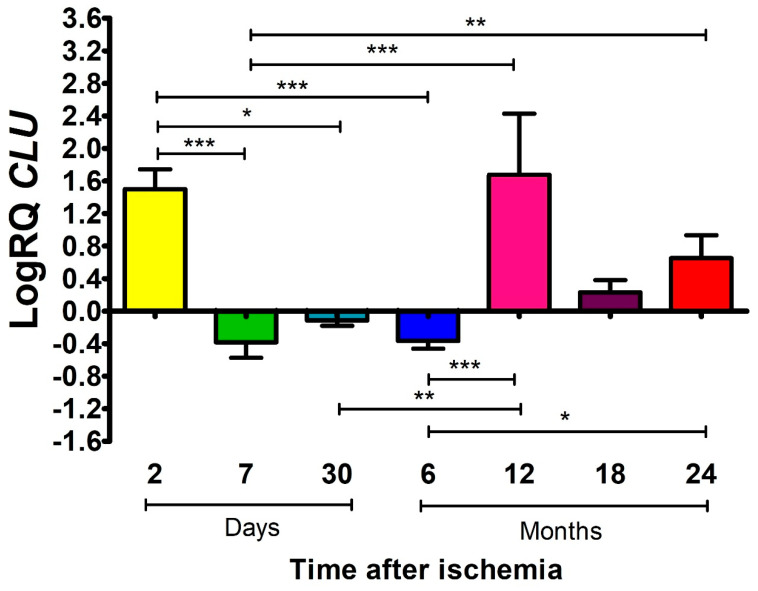
Mean expression levels of *CLU*/*ApoJ* in the frontal cortex. Expression was assessed after 2 (n = 10), 7 (n = 9) and 30 (n = 6) days and after 6 (n = 10), 12 (n = 10), 18 (n = 10) and 24 (n = 10) months after cerebral ischemia. Bars represent (SD) standard deviation. The Kruskal–Wallis test was followed by the “z” test for multiple comparisons. * *p* ≤ 0.05, ** *p* ≤ 0.01, *** *p* ≤ 0.001.

The *ApoJ* encodes the apolipoprotein J. Apolipoprotein J is believed to have beneficial or detrimental effects on the post-ischemic brain, depending on survival time. Two days post-ischemia, the median expression showed a 1.569-fold increase with a minimum/maximum value showing a 1.207/1.931-fold increase and a mean change of 1.500 ± 0.243. An uninterrupted decrease in *ApoJ* expression was noted after 7 and 30 days and 0.5 years, with median values showing changes of −0.410 (minimum −0.594 and maximum −0.084), −0.130 (minimum −0.200 and maximum −0.015), and −0.341 (minimum −0.560 and maximum −0.275), respectively. At the above-presented times, the mean expression values were as follows: −0.383 ± 0.189, −0.114 ± 0.067, and −0.366 ± 0.096. The maximum level of *ApoJ* expression was recorded after 1 year, with a median change of 1.975, and the minimum and maximum values changed by 0.646 and 2.651, respectively, with a mean change of 1.675 ± 0.751. After 1.5 and 2 years from the onset of ischemia, persistent overexpression was observed but at lower levels, the medians showed changes of 0.207 and 0.554, and the minimum and maximum values changed by 0.058/0.565 and 0.346/1.133, with the means showing changes of 0.231 ± 0.150 and 0.651 ± 0.282, respectively. Figure 3 shows the mean values of *ApoJ* expression and statistically significant differences between the times after ischemia.

## 3. Discussion

Our experimental studies showed the overexpression of *ApoA1* in the frontal cortex 7–30 days and 1.5–2 years after an episode of cerebral ischemia. However, after 2 days and in the period of 0.5–1 year after the restoration of cerebral circulation, a decrease in the expression of this gene was observed. Regarding *ApoE* expression, it was lower than the control values from day 7 to 0.5 years, but higher than the control values at the remaining survival times. Moreover, *ApoJ* expression was higher than the control values 2 days and 1–2 years after ischemia; in the remaining recirculation times, it was lower compared to the control values. It should be noted that the pattern of changes in the expression of *ApoE* and *ApoJ* was identical, but completely different from that in *ApoA1*. In this article, we highlight the cross-interactions of amyloid with the studied apolipoprotein genes, which are presented as an integrated part of the amyloid neurotoxicity process, or conversely, as a preventive role in the pathogenesis of Alzheimer’s disease and post-ischemic brain neurodegeneration, especially through the direct binding of apolipoprotein A1 to amyloid [61].

In the cerebral ischemia model used, previous studies have shown increased staining of cortical neurons for ApoA1 during 7–30 days of survival, which currently correlates positively with gene expression [55]. It should be noted that in recent years, ApoA1 has been increasingly recognized as a protective factor against cardiovascular and cerebrovascular diseases [60]. This is supported by findings that elevated ApoA1 levels lower the risk of ischemic stroke, due to its ability to reduce amyloid toxicity and oxidative stress and inhibit neuroinflammatory pathways, which are key mechanisms contributing to vascular health after ischemia [60]. Despite this, it has been shown that cerebral amyloid angiopathy (CAA) develops after ischemia, characterized by the accumulation of amyloid in the walls of cerebral blood vessels [8,15,62], leading to vessel fragility and an increased risk of bleeding complications [60,63]. Recent studies indicate a potential role of ApoA1 in CAA, with particular emphasis on its ability to support the clearance of amyloid from the brain, among others, after ischemia and in Alzheimer’s disease [60,63]. Studies have shown that *ApoA1* deficiency has been associated with increased cortical amyloid deposition and neuroinflammation and that it exacerbates CAA and impairs cognitive function, highlighting its protective effect against amyloid deposition [60,63]. In contrast, the overexpression of *ApoA1* in mice was associated with reduced CAA severity, neuroinflammation, and improved cognitive function [60,63]. Furthermore, in vitro studies have shown that ApoA1 crosses the blood–brain barrier and thus has a strong effect on the outflow of amyloid from the brain [64]. This suggests that ApoA1 may be a therapeutic target for the prevention and treatment of post-ischemic CAA. Although the exact mechanisms by which ApoA1 affects CAA are not fully understood, its potential as a therapeutic target is promising.

ApoA1 has been shown to reduce the number and growth of amyloid fibrils by remodeling them [64]. ApoA1 has been presented to prevent the formation of high-molecular-weight amyloid 1-42 aggregates and reduce amyloid 1-42 toxicity in primary brain cells [60]. Furthermore, the inhibition of amyloid 1-42 aggregation was found to be dependent on the ApoA1 concentration [65]. Moreover, in vitro studies have shown that ApoA1 has a strong affinity for amyloid precursor protein and amyloid 1-40 and inhibits β-sheet formation and amyloid-induced cytotoxicity [66]. ApoA1 interferes with amyloid-induced lipid peroxidation, and in the presence of ApoA1, amyloid aggregates are less neurotoxic than pure amyloid fibrils [67]. As can be seen, all this evidence supports the beneficial role of ApoA1 in limiting amyloid aggregation and toxicity through a direct interaction and complex formation with amyloid. However, the mechanism of this protective effect has not yet been fully elucidated.

ApoA1 has been shown to prevent amyloid cytotoxicity on brain endothelial cells [68]. In transgenic mice, chronic intravenous treatment with ApoA1 Milano resulted in reduced levels of soluble, insoluble, and membrane-bound amyloid 1-42 and 1-40 in the brain [68]. ApoA1 levels have also been found to be lower in patients with Alzheimer’s disease compared to the control group. Furthermore, a correlation has been demonstrated between reduced plasma ApoA1 levels and the occurrence and severity of Alzheimer’s disease [69]. In contrast, high levels of ApoA1 are associated with a reduced risk of dementia in older people [70]. Moreover, *ApoA1*/*ApoE* KO mice showed increased plasma amyloid 1-42 levels and exacerbated memory impairment, suggesting an important role of more than one apolipoprotein in the clearance of amyloid 1-42 from the brain [71].

On the other hand, treatment with human ApoA1 in a transgenic model of Alzheimer’s disease reduced amyloid accumulation, brain neuronal loss, and neuroinflammatory response, and improved cognitive function [68,72]. In contrast, transgenic mice (APP/PS1) lacking ApoA1 showed increased amyloid deposition and astrogliosis in the cerebral cortex, as well as the development of cerebral amyloid angiopathy [63]. Therefore, as shown in these and previous studies, increased *ApoA1* expression after ischemia induces neuroprotective mechanisms [51].

*ApoE* overexpression in the frontal cortex after ischemia was observed on day 2 and after 1–2 years. In the studied model of cerebral ischemia, intra- and extracellular ApoE deposits were present in the cerebral cortex 3 h after ischemia and were often visible even a year later [27,39,55]. Two days after ischemia, massive neuronal degeneration developed with strong staining for ApoE, which correlates well with *ApoE* expression [39]. Importantly, within 1–2 years of ischemic injury, chronic neuronal changes occur, accompanied by acute neuronal changes, and these changes correlate well with *ApoE* expression 1–2 years after ischemia [8,40]. Post-ischemic ApoE staining in the cerebral cortex was detected after short-term and long-term survival in neurons, as well as extracellularly in perivascular area [8,39]. Perivascular ApoE deposits were revealed to co-localize with amyloid precursor protein epitopes after ischemia [55]. In contrast, *ApoE* knockout mice showed significant reductions in amyloid deposits and plaques in a model of amyloidosis [73]. The above observations reveal that the overexpression of *ApoE* may have harmful effects.

The overexpression of *ApoJ* in the frontal cortex was demonstrated 2 days and 1–2 years after ischemia. In our model of cerebral ischemia, intracellular and extracellular ApoJ deposits in the cerebral cortex were observed within 7 days after ischemia [55]. Of note, extracellular perivascular ApoJ deposits in a co-localized manner with post-ischemic amyloid precursor protein epitopes [55]. Historically, ApoJ has been attributed with beneficial effects, including its influence on apoptosis and its activity as a stress-relieving chaperone [74,75]. Furthermore, ApoJ is believed to bind to amyloid and influence its deposition, clearance, and fibrinogenesis, suggesting its key role in the regulation of amyloid metabolism [76,77]. Maximum *ApoJ* expression 1 year after ischemia is associated with increasing and progressive neuronal death and ineffective elimination of the negative effects of ApoE activity [8,27,39,40,55]. However, as seen above, this natural protection is ineffective and inefficient after ischemia, with long survival times. As previously demonstrated, during this time, dying neurons overlapped with degenerating neurons, and this phenomenon became more intense and diffuse over time [8,27,39]. The progression of neurodegenerative processes in the frontal cortex was also associated with persistent changes in the permeability of the blood–brain barrier, starting immediately after the resumption of recirculation and lasting for 2 years after ischemia [8,35,78,79]. Changes in blood–brain barrier permeability were generalized and widespread throughout the brain, with a predominance in the cerebral cortex [35,40,78,79]. Additionally, the above changes in the studied model were accompanied by neuroinflammatory changes during the 2-year follow-up after ischemia [80,81]. Finally, visible atrophy of the cerebral cortex became a fact [8,39,40].

In an experimental model of cerebral ischemia, it was shown that stained ApoJ deposits in the cortex, co-localized with post-ischemic amyloid precursor protein epitopes [55,75,82]. Studies have shown that ApoJ can bind to hyperphosphorylated tau protein, inhibiting its hydrolysis in lysosomes, which leads to tau protein aggregation and causes cell death [83]. In the brains of *ApoJ* knockout mice with ischemia, the lesion area was smaller than in control mice, suggesting that ApoJ does not promote neuronal survival after ischemia [84].

Staining of brain tissue from Alzheimer’s disease patients showed that ApoJ co-localized with amyloid, indicating an interaction between the two [83]. Furthermore, intracellular and secreted ApoJ levels have been shown to increase early in the course of Alzheimer’s disease and are associated with amyloid and tau protein pathology [85]. Additionally, recent research on Alzheimer’s disease also indicates that ApoJ is involved in the formation of amyloid plaques and the progression of the disease [83,86,87]. In addition, ApoJ may influence the progression of Alzheimer’s disease by regulating neuroinflammation, controlling cell apoptosis, and removing pathological proteins [87]. Therefore, it has recently been suggested that ApoJ may promote the occurrence and progression of Alzheimer’s disease [83,86,87,88].

In summary, the pattern of *ApoE* and *ApoJ* expression changes in the frontal cortex was a mirror image. These gene expression changes accompany increasing and progressive neuronal damage in the frontal cortex, encompassing acute and chronic changes that ultimately lead to neuronal death [8,27,39,55,73]. Moreover, extracellular ApoE/ApoJ deposits have been shown to co-localize with amyloid precursor protein epitopes after ischemia [55]. It is worth emphasizing that the expression pattern of *ApoA1* was completely different compared to *ApoE* and *ApoJ* with dominant overexpression 2 years after ischemia. In our studies on genetic changes, the influence of the age at 2 years after ischemia should also be taken into account.

Our research has its strengths and weaknesses. We used female rats in our studies because a significantly higher percentage of them survived ischemia and post-ischemic periods. All animals that were resuscitated after ischemia survive without any problems for 2 years. This observation coincided with the fact that Alzheimer’s disease affects more women than men. The advantages include the survival of animals for up to 2 years after ischemia. Characterizing the ischemic model of Alzheimer’s disease in the context of long-term survival allows for a better understanding of the sequence of spontaneous, unforced changes in the etiology of ischemic Alzheimer’s disease. The ability of animals to survive for up to 2 years in this model enables continuous, long-term observation of neurodegenerative processes associated with Alzheimer’s disease at the genomic, proteomic, structural, functional, neuropathophysiological, neuropathological and behavioral levels. Long-term survival of rats after ischemia increases the accuracy of assessing changes in the studied genes, shows the sequence of changes, and enables mapping their behavior in various brain structures. Experimental studies have shown that early Alzheimer’s disease neuropathology was less severe in young animals that had amyloid plaques, but their volume and density were negligible [11]. It should be emphasized that with age, this advantage disappears and unfavorable genetic changes intensify [11]. The age of the animals participating in our study after ischemia ranged from 2 days to 2 years, which allowed us to follow the development of Alzheimer’s disease-like pathology from early to advanced stages. In future work, we will address the presented problem from a gender perspective, i.e., we will conduct this type of research on male rats. 

The main limitation of our study was the small number of animals in each group. The small number of animals at each time point was due to restrictions imposed by the Bioethics Committee. Moreover, the time of experiments has an impact, i.e., survival up to two years after ischemia, which in humans corresponds to 80 years. This, of course, limited the availability of research material. It should be noted that the presence of apolipoproteins has already been demonstrated in our previous works, in the same model of cerebral ischemia cited in references [27,39,55]. Apolipoproteins were found to localize to neurons and extracellular spaces after cerebral ischemia in animals surviving up to 1 year [27,39,55]. We believe, and we demonstrate this in our work, that gene expression originates in neurons. However, to precisely determine this and ultimately clarify which cells are responsible for the expression, it would be necessary to conduct studies on single cells after ischemia. We are considering researching this in the future.

## 4. Materials and Methods

Wistar female rats (n = 65, 140–160 g) were used to develop a 10 min complete cerebral ischemia with 2-year survival [89]. Sham groups (n = 65) were used as controls. The experimental animals were in a cage (in pairs of 2) in the animal house of 22 ± 2 °C, with 50 ± 5% humidity, with a 12 h dark and light cycle. Rats had unlimited access to food and water during the experiment. The animals used were treated in accordance with the NIH Guide for Care and Use of Laboratory Animals (1985), European Communities Council Directive 142 (86/609/EEC), and with the approval of the local Ethical Committee (No. 53/2014 of 16 January 2015). The rats in ischemic and sham groups were survived following procedure 2 (n = 10), 7 (n = 9), and 30 (n = 6) days and 0.5 (n = 10), 1 (n = 10), 1.5 (n = 10) and 2 (n = 10) years.

After all experiments, the brains were perfused with 0.9% NaCl via the heart. Next, brains were removed from the skull, and ischemic and control samples of about 1 mm^3^ of the frontal cortex were taken. Samples were located in RNALater solution (Life Technologies, Carlsbad, CA, USA). Gene expression of apolipoproteins A1, E and J was evaluated by reverse-transcription quantitative PCR (RT-qPCR) [90].

RNA was obtained from the frontal cortex as a result of homogenization in 1 mL TRI-Reagent buffer (Ambion, Austin, TX, USA). Then, the suspension was incubated for 5 min at room temperature; in the next step, 200 µL of chloroform (Sigma-Aldrich, St. Louis, MO, USA) was added and shaken for 15 s. After that, the contents were incubated for 15 min at room temperature and, after all this, were centrifuged for 15 min at 14,000 rpm. Next to the aqueous part, 500 µL 2-propanol (Sigma-Aldrich, St. Louis, MO, USA) was imparted. The contents were mixed and incubated for 20 min at room temperature. After that, the samples were centrifuged for 20 min at 14,000 rpm at 4 °C. The RNA fraction was washed by 80% ethanol and, finally, RNA was kept in 80% ethanol at −20 °C for investigation. The RNA purity and concentration were rated by spectrophotometry on NanoDrop 2000 (Thermo Scientific, Waltham, MA, USA) [90].

For the main investigations, 1 μg of RNA was reverse-transcribed into cDNA using a high-capacity cDNA kit for reverse transcription (Applied Biosystems, Foster City, CA, USA). The cDNA synthesis was completed on Veriti Dx (Applied Biosystems, Foster City, CA, USA) under the subsequent conditions: stage I 25 °C, 10 min; stage II 37 °C, 120 min; stage III 85 °C, 5 min; stage IV 4 °C. The cDNA, obtained through this formula, was amplified by real-time gene expression analysis (qPCR) on a 7900HT Real-Time Fast System (Applied Biosystems, Foster City, CA, USA) [90].

The studied genes in ischemic and sham samples were evaluated against the control gene Rpl13a. The relative quantity (RQ) of the investigated genes was determined using ΔCT, and the values were presented as RQ = 2^−ΔΔCT^ [90]. The final data were then compiled using a logarithmic conversion of the RQ values (LogRQ) [90]. LogRQ = 0 means that the studied genes have not changed in relation to the control. LogRQ < 0 indicates diminished gene expression and LogRQ > 0 shows the overexpression of genes post-ischemia in relation to the control. Statistica v. 12 was employed to assess the results using the non-parametric Kruskal–Wallis test with the z-test for multiple analyses of differences between groups. The results are showed as mean ± SD. *p* ≤ 0.05 was used to determine statistical significance.

## 5. Conclusions

Current and previously collected data indicate that cerebral ischemia leads to significant dysregulation of Alzheimer’s disease-related genes in various brain structures, including the frontal cortex [6,51,91,92]. As shown above, changes in the expression of apolipoprotein genes occurred from 2 days to 2 years after ischemia and were closely related to the mechanisms of progressive post-ischemia neurodegeneration [6,15,16,80,81,91,93]. Furthermore, cerebral ischemia in rats induced progressive and irreversible cognitive impairment characteristic of Alzheimer’s disease [41,42,43,44]. Also, cerebral ischemia in rats caused excessive amyloid accumulation in the frontal cortex [6,8,91,94].

The data indicate that increased *ApoA1* expression is likely associated with the inhibition of amyloid genesis and/or neuroprotection. In light of the latest data, *ApoJ* expression indicates a dual behavior: a protective effect in the early period after ischemia, but it is detrimental in the long term. However, the overexpression of *ApoE* may influence pro-amyloidogenic and/or neurodegenerative activity. Finally, the *ApoA1* and *ApoJ* products seem to participate in compensating the negative effects of the *ApoE* product as a natural protection, but this protection is ineffective and inefficient once the ischemic phenomenon initiates neurodegeneration.

There is a high probability that the genes studied in this and our other studies could serve as therapeutic targets in preventing or treating the development of neurodegenerative changes after ischemia [6,91]. For example, *ApoA1* is also of interest in the context of human cerebral ischemia, where it is seen as a promising biomarker as well as a potential therapeutic target [95]. Future studies should answer the question of whether *ApoA1* may also be associated with short-term and long-term prognosis in patients after cerebral ischemia, and whether it may be a therapeutic target for preventing recurrent cerebral ischemia. This molecule may also become a valuable target for personalized therapies, which can be expected in the near future. It is clear that studying the cross-interaction of the protective ApoA1 with amyloid 1–42 would clarify certain phenomena and mechanisms of ApoA1 action. In particular, the crystallographic structure of ApoA1 is already known [96], but there is still no information on the crystal structure of the amyloid-ApoA1 complex. Further studies are needed to clarify the role of ApoA1 in CAA and its impact on amyloid deposition and cognitive outcomes. Therefore, studying the effect of ApoA1 on vascular integrity and amyloid clearance may provide new therapeutic options for CAA [60].

Therefore, further research on the relationship between *ApoJ* and cerebral ischemia and Alzheimer’s disease will contribute to a deeper understanding of the etiology of both neurodegenerative diseases and provide a theoretical basis for the development of early diagnostic and therapeutic strategies in these cases. Furthermore, *ApoJ* may act in conjunction with other genetic risk factors, such as *ApoE,* and this in turn may play a role in the development and progression of post-ischemic neurodegeneration and Alzheimer’s disease [97]. In summary, the role of *ApoJ* in Alzheimer’s disease and ischemic neurodegeneration is complex and not fully understood. Thus, further studies are needed to understand the transcription patterns of *ApoJ* in various cell types, brain areas, during development and aging, in the healthy brain and in disease, and in individuals of different sexes.

One of the key directions of future research is to precisely understand the dynamics of temporal changes in various cells of the genes we studied in neurodegeneration after ischemia and Alzheimer’s disease. Sequencing single cells after a pathological episode would reveal significant changes in gene expression at different stages of the disease and would allow for an understanding of how these changes evolve over time in relation to disease onset and progression. Long-term studies using single-cell technologies in post-ischemia and Alzheimer’s disease may provide deeper insights into the timing and sequence of cellular dysfunction, enabling a more precise identification of early biomarkers and potential therapeutic windows.

Another important subject for future research is to understand the role of the brain microenvironment in post-ischemic neurodegeneration and Alzheimer’s disease. Although we know that neuronal and neuroglial cells as well as blood vessels play distinct roles in the progression of both diseases, the interactions between these cells and their modulation by the adjacent microenvironment are not fully understood at present. Advances in spatial transcriptomics combined with single-cell analysis offer hope for understanding how the local brain environment influences cell behavior and disease progression.

Furthermore, although genetic and epigenetic changes in post-ischemic neurodegeneration and Alzheimer’s disease have been well-recognized, the exact processes by which these alterations affect progress of diseases remain unclear. It is therefore unknown how exact genetic variants or epigenetic alterations interact with environmental brain components to cause the heterogeneity noted in patients with brain ischemia and Alzheimer’s disease. In summary, concentrating on the molecular diversity of cerebral cells and the dynamic alterations in gene expression in miscellaneous cells will allow us to achieve significant progress in overcoming the limitations of traditional methods of studying brain tissue. This type of work will help define the role of neuronal and neuroglial cells and blood vessels in the progression of cerebral ischemia and Alzheimer’s disease, and will also provide important facts on the complex interactions in the brain microenvironment—a problem that has not been researched yet. These proposals open new possibilities for explaining the development of post-ischemic brain neurodegeneration and Alzheimer’s disease and may reveal new therapeutic interventions aimed at restoring brain homeostasis, in particular by targeting specific cell types, molecular pathways, and the brain tissue microenvironment disturbed by ischemia and Alzheimer’s disease.

## Data Availability

The data of this study can be made available on request from the corresponding author.

## Abbreviations

The following abbreviations are used in this manuscript:

ApoA1	Apolipoprotein A1
ApoE	Apolipoprotein E
ApoJ	Apolipoprotein J
Apos	Apolipoproteins
CLU	Clusterin
CAA	Cerebral amyloid angiopathy

## References

[1] Kamatham P.T., Shukla R., Khatri D.K., Vora L.K. (2024). Pathogenesis, diagnostics, and therapeutics for Alzheimer’s disease: Breaking the memory barrier. Ageing Res. Rev..

[2] Behl T., Kaur I., Sehgal A., Khandige P.S., Imran M., Gulati M., Khalid Anwer M., Elossaily G.M., Ali N., Wal P. (2024). The link between Alzheimer’s disease and stroke: A detrimental synergism. Ageing Res. Rev..

[3] Dammavalam V., Rupert D., Lanio M., Jin Z., Nadkarni N., Tsirka S.E., Bergese S.D. (2024). Dementia after Ischemic Stroke, from Molecular Biomarkers to Therapeutic Options. Int. J. Mol. Sci..

[4] Hou S., Zhang Y., Xia Y., Liu Y., Deng X., Wang W., Wang Y., Wang C., Wang G. (2024). Global regional national epidemiology of ischemic stroke from 1990 to 2021. Eur. J. Neurol..

[5] Ngamdu K.S., Kalra D.K. (2024). Risk of Stroke, Dementia, and Cognitive Decline with Coronary and Arterial Calcification. J. Clin. Med..

[6] Pluta R. (2024). A look at the etiology of Alzheimer’s disease based on the brain ischemia model. Curr. Alzheimer Res..

[7] Almeida Z.L., Vaz D.C., Brito R.M.M. (2025). Morphological and Molecular Profiling of Amyloid-β Species in Alzheimer’s Pathogenesis. Mol. Neurobiol..

[8] Pluta R., Ułamek M., Jabłoński M. (2009). Alzheimer’s mechanisms in ischemic brain degeneration. Anat. Rec..

[9] Pluta R., Januszewski S., Czuczwar S.J. (2021). Brain Ischemia as a Prelude to Alzheimer’s Disease. Front. Aging Neurosci..

[10] Elman-Shina K., Efrati S. (2022). Ischemia as a common trigger for Alzheimer’s disease. Front. Aging Neurosci..

[11] Lecordier S., Pons V., Rivest S., ElAli A. (2022). Multifocal Cerebral Microinfarcts Modulate Early Alzheimer’s Disease Pathology in a Sex-Dependent Manner. Front. Immunol..

[12] Pluta R. (2022). Brain ischemia as a bridge to Alzheimer’s disease. Neural. Regen. Res..

[13] Das T.K., Ganesh B.P., Fatima-Shad K. (2023). Common signaling pathways involved in Alzheimer’s disease and stroke: Two faces of the same coin. J. Alzheimer’s Dis. Rep..

[14] Li X.Y., Quan M., Wei Y., Wang W., Xu L., Wang Q., Jia J. (2023). Critical thinking of Alzheimer’s transgenic mouse model: Current research and future perspective. Sci. China-Life Sci..

[15] Pluta R. (2025). Neuroinflammation in the Post-Ischemic Brain in the Presence of Amyloid and Tau Protein. Discov. Med..

[16] Pluta R. (2025). Direct and indirect role of non-coding RNAs in company with amyloid and tau protein in promoting neuroinflammation in post-ischemic brain neurodegeneration. Front. Cell Neurosci..

[17] Singh S.K., Srivastav S., Yadav A.K., Srikrishna S., Perry G. (2016). Overview of Alzheimer’s disease and some therapeutic approaches targeting Aβ by using several synthetic and herbal compounds. Oxid. Med. Cell. Longev..

[18] Nasb M., Tao W., Chen N. (2024). Alzheimer’s Disease Puzzle: Delving into Pathogenesis Hypotheses. Aging Dis..

[19] Lazarov O., Gupta M., Kumar P., Morrissey Z., Phan T. (2024). Memory Circuits in Dementia: The Engram, Hippocampal Neurogenesis and Alzheimer’s Disease. Prog. Neurobiol..

[20] Qian Z., Li Y., Ye K. (2024). Advancements and challenges in mouse models of Alzheimer’s disease. Trends Mol. Med..

[21] Pádua M.S., Guil-Guerrero J.L., Prates J.A.M., Lopes P.A. (2024). Insights on the Use of Transgenic Mice Models in Alzheimer’s Disease Research. Int. J. Mol. Sci..

[22] Zhong M.Z., Peng T., Duarte M.L., Wang M., Cai D. (2024). Updates on mouse models of Alzheimer’s disease. Mol. Neurodegener..

[23] Castellani R.J., Jamshidi P., Plascencia-Villa G., Perry G. (2025). The Amyloid Cascade Hypothesis: A Conclusion in Search of Support. Am. J. Pathol..

[24] Arruda B.P., Martins P.P., Kihara A.H., Takada S.H. (2025). Perinatal asphyxia and Alzheimer’s disease: Is there a correlation?. Front. Pediatr..

[25] Hardy J. (2025). Alzheimer’s Disease: Treatment Challenges for the Future. J. Neurochem..

[26] Pluta R., Kida E., Lossinsky A.S., Golabek A.A., Mossakowski M.J., Wisniewski H.M. (1994). Complete cerebral ischemia with short-term survival in rats induced by cardiac arrest. I. Extracellular accumulation of Alzheimer’s beta-amyloid protein precursor in the brain. Brain Res..

[27] Pluta R., Barcikowska M., Debicki G., Ryba M., Januszewski S. (1997). Changes in amyloid precursor protein and apolipoprotein E immunoreactivity following ischemic brain injury in rat with long-term survival: Influence of idebenone treatment. Neurosci. Lett..

[28] van Groen T., Puurunen K., Mäki H.M., Sivenius J., Jolkkonen J. (2005). Transformation of diffuse beta-amyloid precursor protein and beta-amyloid deposits to plaques in the thalamus after transient occlusion of the middle cerebral artery in rats. Stroke.

[29] Qi J.P., Wu H., Yang Y., Wang D.D., Chen Y.X., Gu Y.H., Liu T. (2007). Cerebral ischemia and Alzheimer’s disease: The expression of amyloid-beta and apolipoprotein E in human hippocampus. J. Alzheimer’s Dis..

[30] Bracko O., Njiru B.N., Swallow M., Ali M., Haft-Javaherian M., Schaffer C.B. (2020). Increasing cerebral blood flow improves cognition into late stages in Alzheimer’s disease mice. J. Cereb. Blood Flow Metab..

[31] Solis E., Hascup K.N., Hascup E.R. (2020). Alzheimer’s disease: The link between amyloid-β and neurovascular dysfunction. J. Alzheimer’s Dis..

[32] Kato T., Hirano A., Katagiri T., Sasaki H., Yamada S. (1988). Neurofibrillary tangle formation in the nucleus basalis of Meynert ipsilateral to a massive cerebral infarct. Ann. Neurol..

[33] Hatsuta H., Takao M., Nogami A., Uchino A., Sumikura H., Takata T., Morimoto S., Kanemaru K., Adachi T., Arai T. (2019). Tau and TDP-43 accumulation of the basal nucleus of Meynert in individuals with cerebral lobar infarcts or hemorrhage. Acta Neuropathol. Commun..

[34] Chung Y.A., Kim J.Y., Kim K.J., Ahn K.J. (2009). Hypoperfusion and ischemia in cerebral amyloid angiopathy documented by 99mTc-ECD brain perfusion SPECT. J. Nucl. Med..

[35] Pluta R., Miziak B., Czuczwar S.J. (2023). Post-ischemic permeability of the blood–brain barrier to amyloid and platelets as a factor in the maturation of Alzheimer’s disease-type brain neurodegeneration. Int. J. Mol. Sci..

[36] Pluta R., Kiś J., Januszewski S., Jabłoński M., Czuczwar S.J. (2022). Cross-Talk between Amyloid, Tau Protein and Free Radicals in Post-Ischemic Brain Neurodegeneration in the Form of Alzheimer’s Disease Proteinopathy. Antioxidants.

[37] Yuan Y., Shan X., Men W., Zhai H., Qiao X., Geng L., Li C. (2020). The effect of crocin on memory, hippocampal acetylcholine level, and apoptosis in a rat model of cerebral ischemia. Biomed. Pharmacother..

[38] Li B., Liu S., Ren T., BoYang Yan B.Y. (2023). Effects of curcumin on memory, hippocampal acetylcholine level and neuroapoptosis in repeated cerebral ischemia rat model. Pak. J. Pharm. Sci..

[39] Pluta R. (2000). The role of apolipoprotein E in the deposition of beta-amyloid peptide during ischemia-reperfusion brain injury. A model of early Alzheimer’s disease. Ann. N. Y. Acad. Sci..

[40] Jabłoński M., Maciejewski R., Januszewski S., Ułamek M., Pluta R. (2011). One year follow up in ischemic brain injury and the role of Alzheimer factors. Physiol. Res..

[41] de la Tremblaye P.B., Plamondon H. (2011). Impaired conditioned emotional response and object recognition are concomitant to neuronal damage in the amygdala and perirhinal cortex in middle-aged ischemic rats. Behav. Brain Res..

[42] Kiryk A., Pluta R., Figiel I., Mikosz M., Ulamek M., Niewiadomska G., Jablonski M., Kaczmarek L. (2011). Transient brain ischemia due to cardiac arrest causes irreversible long-lasting cognitive injury. Behav. Brain Res..

[43] Li J., Wang Y.J., Zhang M., Fang C.Q., Zhou H.D. (2011). Cerebral ischemia aggravates cognitive impairment in a rat model of Alzheimer’s disease. Life Sci..

[44] Cohan C.H., Neumann J.T., Dave K.R., Alekseyenko A., Binkert M., Stransky K., Lin H.W., Barnes C.A., Wright C.B., Perez-Pinzon M.A. (2015). Effect of cardiac arrest on cognitive impairment and hippocampal plasticity in middle-aged rats. PLoS ONE.

[45] Deng L., Gupta V.K., Wu Y., Pushpitha K., Chitranshi N., Gupta V.B., Fitzhenry M.J., Moghaddam M.Z., Karl T., Salekdeh G.H. (2021). Mouse model of Alzheimer’s disease demonstrates differential effects of early disease pathology on various brain regions. Proteomics.

[46] Endres K. (2021). Apolipoprotein A1, the neglected relative of Apolipoprotein E and its potential role in Alzheimer’s disease. Neural. Regen. Res..

[47] Raulin A.C., Martens Y.A., Bu G. (2022). Lipoproteins in the Central Nervous System: From Biology to Pathobiology. Annu. Rev. Biochem..

[48] Islam S., Noorani A., Sun Y., Michikawa M., Zou K. (2025). Multi-functional role of apolipoprotein E in neurodegenerative diseases. Front. Aging Neurosci..

[49] Chinnathambi S., Adityan A., Chidambaram H., Chandrashekar M. (2025). Apolipoprotein E and Tau interaction in Alzheimer’s disease. Adv. Protein Chem. Struct. Biol..

[50] Juul Rasmussen I., Luo J., Frikke-Schmidt R. (2024). Lipids, lipoproteins, and apolipoproteins: Associations with cognition and dementia. Atherosclerosis.

[51] Pluta R., Kocki J., Bogucki J., Bogucka-Kocka A., Czuczwar S.J. (2025). Apolipoprotein (*APOA1*, *APOE*, *CLU*) genes expression in the CA3 region of the hippocampus in an ischemic model of Alzheimer’s disease with survival up to 2 years. J. Alzheimer’s Dis..

[52] Kalani R., Krishnamoorthy S., Deepa D., Gopala S., Prabhakaran D., Tirschwell D., Sylaja P.N. (2020). Apolipoproteins B and A1 in Ischemic Stroke Subtypes. J. Stroke Cerebrovasc. Dis..

[53] Rhea E.M., Banks W.A. (2021). Interactions of lipids, lipoproteins, and apolipoproteins with the blood–brain barrier. Pharm. Res..

[54] Etuze T., Triniac H., Zheng Z., Vivien D., Dubois F. (2025). Apolipoproteins in ischemic stroke progression and recovery: Key molecular mechanisms and therapeutic potential. Neurobiol. Dis..

[55] Kida E., Pluta R., Lossinsky A.S., Golabek A.A., Choi-Miura N.H., Wisniewski H.M., Mossakowski M.J. (1995). Complete cerebral ischemia with short-term survival in rat induced by cardiac arrest. II. Extracellular and intracellular accumulation of apolipoproteins E and J in the brain. Brain Res..

[56] Chemello K., Guedon A.F., Techer R., Jaafar A.K., Guilhen S., Swietek M.J., Lambert G., Gallo A. (2025). Apolipoprotein E Plasma Concentrations Are Predictive of Recurrent Strokes: Insights From the SPARCL Trial. J. Am. Heart. Assoc..

[57] Liang N., Jiang F., Wang L., Liu X., Jia R., Liu M., Hu J. (2025). The hypoperfusion intensity ratio associates with APOE gene polymorphism in acute ischemic stroke patients with large vessel occlusion. Sci. Rep..

[58] Liu J., Xu Z., Wen Y., Guo X., Chen X., Liu D., Li L., Liu H. (2025). Apolipoprotein A1 and Lipoprotein(a) as Biomarkers for the “Penumbra Freezing” in Acute Ischemic Stroke: Insights From a Case-Control and Mendelian Randomization Study. Curr. Med. Chem..

[59] Pluta R., Kocki J., Bogucka-Kocka A., Bogucki J., Czuczwar S.J. (2025). Alterations of Mitophagy (BNIP3), Apoptosis (CASP3), and Autophagy (BECN1) Genes in the Frontal Cortex in an Ischemic Model of Alzheimer’s Disease with Long-Term Survival. Curr. Alzheimer’s Res..

[60] Rajha H.E., Hassanein A., Mesilhy R., Nurulhaque Z., Elghoul N., Burgon P.G., Al Saady R.M., Pedersen S. (2025). Apolipoprotein A (ApoA) in Neurological Disorders: Connections and Insights. Int. J. Mol. Sci..

[61] Ciccone L., Shi C., di Lorenzo D., Van Baelen A.C., Tonali N. (2020). The Positive Side of the Alzheimer’s Disease Amyloid Cross-Interactions: The Case of the Abeta 1-42 Peptide with Tau, TTR, CysC, and ApoA1. Molecules.

[62] Rost N.S., Brodtmann A., Pase M.P., van Veluw S.J., Biffi A., Duering M., Hinman J.D., Dichgans M. (2022). Post-Stroke Cognitive Impairment and Dementia. Circ. Res..

[63] Button E.B., Boyce G.K., Wilkinson A., Stukas S., Hayat A., Fan J., Wadsworth B.J., Robert J., Martens K.M., Wellington C.L. (2019). ApoA-I deficiency increases cortical amyloid deposition, cerebral amyloid angiopathy, cortical and hippocampal astrogliosis, and amyloid-associated astrocyte reactivity in APP/PS1 mice. Alzheimer’s Res. Ther..

[64] Dal Magro R., Simonelli S., Cox A., Formicola B., Corti R., Cassina V., Nardo L., Mantegazza F., Salerno D., Grasso G. (2019). The Extent of Human Apolipoprotein A-I Lipidation Strongly Affects the β-Amyloid Effux Across the Blood–brain barrier in vitro. Front. Neurosci..

[65] Lefterov I., Fitz N.F., Cronican A.A., Fogg A., Lefterov P., Kodali R., Wetzel R., Koldamova R. (2010). Apolipoprotein A-I Deficiency Increases Cerebral Amyloid Angiopathy and Cognitive Deficits in APP/PS1DE9 Mice. J. Biol. Chem..

[66] Koldamova R.P., Lefterov I.M., Lefterova M.I., Lazo J.S. (2001). Apolipoprotein A-I Directly Interacts with Amyloid Precursor Protein and Inhibits Aβ Aggregation and Toxicity. Biochemistry.

[67] Paula-Lima A.C., Tricerri M.A., Brito-Moreira J., Bomfim T.R., Oliveira F.F., Magdesian M.H., Grinberg L.T., Panizzutti R., Ferreira S.T. (2009). Human apolipoprotein A–I binds amyloid- and prevents A-induced neurotoxicity. Int. J. Biochem. Cell Biol..

[68] Fernández-de Retana S., Montañola A., Marazuela P., De La Cuesta M., Batlle A., Fatar M., Grudzenski S., Montaner J., Hernández-Guillamon M. (2017). Intravenous treatment with human recombinant ApoA-I Milano reduces beta amyloid cerebral deposition in the APP23-transgenic mouse model of Alzheimer’s disease. Neurobiol. Aging.

[69] Merched A., Xia Y., Visvikis S., Serot J.M., Siest G. (2000). Decreased high-density lipoprotein cholesterol and serum apolipoprotein AI concentrations are highly correlated with the severity of Alzheimer’s disease. Neurobiol. Aging.

[70] Saczynski J.S., White L., Peila R.L., Rodriguez B.L., Launer L.J. (2007). The Relation between Apolipoprotein A-I and Dementia. The Honolulu-Asia Aging Study. Am. J. Epidemiol..

[71] Fitz N.F., Tapias V., Cronican A.A., Castranio E.L., Saleem M., Carter A.Y., Lefterova M., Lefterov I., Koldamova R. (2015). Opposing effects of Apoe/Apoa1 double deletion on amyloid-β pathology and cognitive performance in APP mice. Brain.

[72] Solé M., Marazuela P., Castellote L., Bonaterra-Pastra A., Giménez-Llort L., Hernández-Guillamon M. (2023). Therapeutic effect of human ApoA-I-Milano variant in aged transgenic mouse model of Alzheimer’s disease. Br. J. Pharmacol..

[73] Mahan T.E., Wang C., Bao X., Choudhury A., Ulrich J.D., Holtzman D.M. (2022). Selective reduction of astrocyte apoE3 and apoE4 strongly reduces Aβ accumulation and plaque-related pathology in a mouse model of amyloidosis. Mol. Neurodegener..

[74] Poon S., Easterbrook-Smith S.B., Rybchyn M.S., Carver J.A., Wilson M.R. (2000). Clusterin is an ATP-independent chaperone with very broad substrate specificity that stabilizes stressed proteins in folding competent state. Biochemistry.

[75] Herring S.K., Moon H.-J., Rawal P., Chhibber A., Zhao L. (2019). Brain clusterin protein isoforms and mitochondrial localization. Elife.

[76] Bell R.D., Sagare A.P., Friedman A.E., Bedi G.S., Holtzman D.M., Deane R., Zlokovic B.V. (2007). Transport pathways for clearance of human Alzheimer’s amyloid beta-peptide and apolipoproteins E and J in the mouse central nervous system. J. Cereb. Blood Flow Metab..

[77] Wojtas A.M., Kang S.S., Olley B.M., Gatherer M., Shinohara M., Lozano P.A., Liu C.C., Kurti A., Baker K.E., Dickson D.W. (2017). Loss of clusterin shifts amyloid deposition to the cerebrovasculature via disruption of perivascular drainage pathways. Proc. Natl. Acad. Sci. USA.

[78] Pluta R., Lossinsky A.S., Wiśniewski H.M., Mossakowski M.J. (1994). Early blood–brain barrier changes in the rat following transient complete cerebral ischemia induced by cardiac arrest. Brain Res..

[79] Pluta R., Januszewski S., Ułamek M. (2008). Ischemic blood–brain barrier and amyloid in white matter as etiological factors in leukoaraiosis. Acta Neurochir. Suppl..

[80] Sekeljic V., Bataveljic D., Stamenkovic S., Ułamek M., Jabłoński M., Radenovic L., Pluta R., Andjus P.R. (2012). Cellular markers of neuroinflammation and neurogenesis after ischemic brain injury in the long-term survival rat model. Brain Struct. Funct..

[81] Radenovic L., Nenadic M., Ułamek-Kozioł M., Januszewski S., Czuczwar S.J., Andjus P.R., Pluta R. (2020). Heterogeneity in brain distribution of activated microglia and astrocytes in a rat ischemic model of Alzheimer’s disease after 2 years of survival. Aging.

[82] Wiggins A.K., Shen P.-J., Gundlach A.L. (2003). Delayed, but prolonged increases in astrocytic clusterin (ApoJ) mRNA expression following acute cortical spreading depression in the rat: Evidence for a role of clusterin in ischemic tolerance. Mol. Brain Res..

[83] Du X., Chen Z., Shui W. (2025). Clusterin: Structure, function and roles in disease. Int. J. Med. Sci..

[84] Han B.H., DeMattos R.B., Dugan L.L., Kim-Han J.S., Brendza R.P., Fryer J.D., Kierson M., Cirrito J., Quick K., Harmony J.A. (2001). Clusterin contributes to caspase-3-independent brain injury following neonatal hypoxia-ischemia. Nat. Med..

[85] Shepherd C.E., Affleck A.J., Bahar A.Y., Carew-Jones F., Halliday G.M. (2020). Intracellular and secreted forms of clusterin are elevated early in Alzheimer’s disease and associate with both Aβ and tau pathology. Neurobiol. Aging.

[86] Liu X., Che R., Liang W., Zhang Y., Wu L., Han C., Lu H., Song W., Wu Y., Wang Z. (2022). Clusterin transduces Alzheimer-risk signals to amyloidogenesis. Signal Transduct. Target. Ther..

[87] Palihati N., Tang Y., Yin Y., Yu D., Liu G., Quan Z., Ni J., Yan Y., Qing H. (2024). Clusterin is a Potential Therapeutic Target in Alzheimer’s Disease. Mol. Neurobiol..

[88] Yuste-Checa P., Trinkaus V.A., Riera-Tur I., Imamoglu R., Schaller T.F., Wang H., Dudanova I., Hipp M.S., Bracher A., Hartl F.U. (2021). The extracellular chaperone Clusterin enhances Tau aggregate seeding in a cellular model. Nat. Commun..

[89] Pluta R., Lossinsky A.S., Mossakowski M.J., Faso L., Wisniewski H.M. (1991). Reassessment of a new model of complete cerebral ischemia in rats. Method of induction of clinical death, pathophysiology and cerebrovascular pathology. Acta Neuropathol..

[90] Kocki J., Ułamek-Kozioł M., Bogucka-Kocka A., Januszewski S., Jabłoński M., Gil-Kulik P., Brzozowska J., Petniak A., Furmaga-Jabłońska W., Bogucki J. (2015). Dysregulation of Amyloid-β Protein Precursor, β-Secretase, Presenilin 1 and 2 Genes in the Rat Selectively Vulnerable CA1 Subfield of Hippocampus Following Transient Global Brain Ischemia. J. Alzheimer’s Dis..

[91] Pluta R., Czuczwar S.J. (2024). Ischemia-reperfusion programming of Alzheimer’s disease-related genes—A new perspective on brain neurodegeneration after cardiac arrest. Int. J. Mol. Sci..

[92] Pluta R., Bogucka-Kocka A., Kocki J., Bogucki J., Czuczwar S.J. (2026). Tau Protein, α-synuclein, and Amyloid Precursor Protein Processing Genes in the Frontal Cortex of an Ischemic Alzheimer’s Disease Model. Curr. Alzheimer Res..

[93] Pluta R., Kocki J., Bogucki J., Bogucka-Kocka A., Czuczwar S.J. (2023). LRP1 And RAGE genes transporting amyloid and tau protein in the hippocampal CA3 area in an ischemic model of Alzheimer’s disease with 2-year survival. Cells.

[94] Babusikova E., Dobrota D., Turner A.J., Nalivaeva N.N. (2021). Effect of global brain ischemia on amyloid precursor protein metabolism and expression of amyloid-degrading enzymes in rat cortex: Role in pathogenesis of Alzheimer’s disease. Biochemistry.

[95] Schreiner T.G., Ignat B.E., Grosu C., Costache A.D., Leon M.M., Mitu F. (2024). Lipid-derived biomarkers as therapeutic targets for chronic coronary syndrome and ischemic stroke: An updated narrative review. Medicina.

[96] Borhani D.W., Rogers D.P., Engler J.A., Brouillette C.G. (1997). Crystal structure of truncated human apolipoprotein A-I suggests a lipid-bound conformation. Proc. Natl. Acad. Sci. USA.

[97] Milinkeviciute G., Green K.N. (2023). Clusterin/apolipoprotein J, its isoforms and Alzheimer’s disease. Front. Aging Neurosci..

